# Clinical characteristics and outcomes in risk-stratified patients with smoldering multiple myeloma: data from the Czech Republic Registry of Monoclonal Gammopathies

**DOI:** 10.1038/s41408-023-00906-7

**Published:** 2023-09-27

**Authors:** Viera Sandecka, Tereza Popkova, Martin Stork, Vladimir Maisnar, Jiri Minarik, Alexandra Jungova, Petr Pavlicek, Lukas Stejskal, Lenka Pospisilova, Adriana Heindorfer, Jarmila Obernauerova, Evzen Gregora, Michal Sykora, Jana Ullrychova, Marek Wrobel, Petr Kessler, Tomas Jelinek, Peter Kunovszki, Sacheeta Bathija, Blanca Gros, Sabine Wilbertz, Qian Cai, Annette Lam, Ivan Spicka

**Affiliations:** 1 University Hospital Brno, Brno, Czech Republic; 2 University Hospital Ostrava and Faculty of Medicine, Ostrava, Czech Republic; 3 Charles University Hospital and Faculty of Medicine Hradec Kralove, Hradec Kralove, Czech Republic; 4 University Hospital Olomouc, Olomouc, Czech Republic; 5 University Hospital Pilsen, Pilsen, Czech Republic; 6 University Hospital Kralovske Vinohrady, Prague, Czech Republic; 7 Silesian Hospital in Opava, Opava, Czech Republic; 8 Institute of Biostatistics and Analyses Ltd, Brno, Czech Republic; 9 Liberec Regional Hospital, Liberec, Czech Republic; 10 Regional Hospital Mlada Boleslav, Mlada Boleslav, Czech Republic; 11 Motol University Hospital, Prague, Czech Republic; 12 Hospital Ceske Budejovice, Ceske Budejovice, Czech Republic; 13 KZ, Masaryk Hospital in Usti nad Labem, Usti nad Labem, Czech Republic; 14 Hospital Novy Jicin, Novy Jicin, Czech Republic; 15 Hospital Pelhrimov, Pelhrimov, Czech Republic; 16 Janssen Global Services, Budapest, Hungary; 17 Janssen Global Services, Raritan, NJ USA; 18 Janssen-Cilag, Madrid, Spain; 19 Janssen-Cilag GmbH, Neuss, Germany; 20 Janssen Global Services, Titusville, NJ USA; 21 Charles University and General Hospital in Prague, Prague, Czech Republic

**Keywords:** Myeloma, Myeloma

## Abstract

Smoldering multiple myeloma (SMM) is an asymptomatic precursor to active multiple myeloma (MM). The aim of this study was to report clinical characteristics and outcomes of patients with SMM stratified based on their risk of progression to MM using the Mayo 20/2/20 criteria. Data were leveraged from the Czech Myeloma Group Registry of Monoclonal Gammopathies (RMG). Key outcomes included progression-free survival from SMM diagnosis to active MM diagnosis or death (PFS), progression-free survival from SMM diagnosis to progression on first line (1 L) MM treatment or death (PFS2), and overall survival (OS). Of 498 patients, 174 (34.9%) were classified as high risk and 324 (65.1%) as non–high risk. Median follow-up was approximately 65 months. During follow-up, more patients in the high-risk vs non–high-risk group received 1 L MM treatment (76.4% vs 46.6%, *p* < 0.001). PFS, PFS2, and OS were significantly shorter in high-risk vs non–high-risk patients (13.2 vs 56.6 months, *p* < 0.001; 49.9 vs 84.9 months, *p* < 0.001; 93.2 vs 131.1 months, *p* = 0.012, respectively). The results of this study add to the growing body of evidence that patients with high-risk vs non–high-risk SMM have significantly worse outcomes, including OS.

## Introduction

Smoldering multiple myeloma (SMM) is a plasma cell disorder that has the potential to progress to active multiple myeloma (MM) [1]. SMM is generally asymptomatic and people are often unaware of having the condition until they are diagnosed during a routine laboratory examination [2]. SMM is defined as serum M protein (IgG or IgA) ≥ 3 g/dl or urinary M protein ≥500 mg/24 h, and/or bone marrow plasma cells (BMPCs) of 10% to 60% and the absence of myeloma defining events (hypercalcemia, anemia, lytic bone lesions, or renal insufficiency) or amyloidosis [3]. SMM is a rare disorder with an estimated incidence in the United States of 0.9 cases per 100,000 people and a median age at diagnosis of approximately 67 years [4]. Data from the Swedish Myeloma Registry, using the world population as reference, have indicated an age-standardized incidence of 0.44 cases per 100,000 [5] and a nationwide Icelandic screening study (iStopMM) demonstrated a prevalence of SMM of approximately 0.5% and was higher in men (0.7%) than women (0.4%) [6]. However, due to the asymptomatic course of the disorder and evolving diagnostic criteria, SMM may be underdiagnosed.

The risk for progression in patients with SMM has been estimated to be 73% over 15 years and to be greatest in the first few years following diagnosis: 10% per year over the first 5 years, 3% per year for the next 5 years, and 1% per year for the following 10 years [7]. However, there is a great deal of variability in outcomes in patients diagnosed with SMM; some patients do not progress at all over the course of their lifetime or progress very slowly to active MM, whereas others experience a quick transition [1, 8, 9].

Outcomes may vary by the risk of progression from SMM to active MM and several models have been developed to risk-stratify patients with SMM. In 2018, the Mayo clinic published a model (20/2/20) that classified patients into low-, intermediate-, and high-risk categories, 9.7%, 26.3%, and 47.4% of whom respectively progressed to active MM at 2 years [10]. This model defines high risk as involved to uninvolved free light chain (FLC) ratio >20, serum M protein >2 g/dl, and BMPC infiltration >20%. More recently, the International Myeloma Working Group (IMWG) validated the Mayo 20/2/20 criteria and evaluated including cytogenetic abnormalities (t(4;14), t(14;16), +1q, and/or del13q/monosomy 13) to the Mayo 20/2/20 criteria, which identified a population of high-risk patients with SMM who had ≥3 risk factors and a 63% risk of progression to MM within 2 years [11]. In 2020, the Czech Myeloma Group developed a risk stratification model that identified a group of ultra-high-risk patients with SMM who were estimated to have an 80% chance of progression to active MM within 2 years using serum parameters (FLC ratio >30, immunoparesis, and serum M protein ≥2.3 g/dl) [12].

Given the increased risk of progression to active MM, subsequent end-organ damage, and risk of death in patients with high-risk SMM [1], there is a need for frequent risk monitoring in all patients with SMM and for the evaluation of early therapeutic intervention in those patients identified as at high risk. There are currently no approved treatments for SMM. The European Hematology Association (EHA) and European Society for Medical Oncology (ESMO) clinical practice guidelines [13] and the National Comprehensive Cancer Network© (NCCN) guidelines [14] recommend observation and entry into clinical trials for patients at high-risk for progression defined by the Mayo 20/2/20 criteria. The NCCN guidelines also recommend off-label treatment with lenalidomide in certain circumstances for patients with high-risk SMM [14].

The aim of this study was to utilize data from the Czech Registry of Monoclonal Gammopathies (RMG) to assess and compare the clinical characteristics and outcomes of patients with SMM who were risk-stratified using the Mayo 20/2/20 criteria into high-risk and non–high-risk (low-/ intermediate-risk) SMM. This study allowed for the investigation of additional outcomes beyond those previously examined, including progression-free survival (PFS) and overall survival (OS) in the European population.

## Methods

### Data source

This study evaluated retrospective data extracted from the RMG. This registry was established by the Czech Myeloma Group and compiles clinical data relating to the diagnosis, treatment, and survival of patients with monoclonal gammopathies [15] and is one of the largest European registries of its kind [16]. While this registry included data on patients with monoclonal gammopathies from 19 hematological centers in the Czech Republic and 4 in Slovakia [17], this study specifically evaluated available data in patients with SMM from 18 of the centers in the Czech Republic from January 1980 to November 2021. The RMG was established in 2007 [15], although some patients were included retrospectively from 1980. All included patients consented to their data being used for research when their data was entered in the registry. The centers consented to the data being used in the study.

### Patients

Patients presenting with ≥1 of the following SMM criteria were included: serum M protein ≥30 g/l, urinary M protein ≥500 mg/24 h, or ≥10% of clonal bone marrow plasma cells (BMPCs). Index date was designated as the date of the first SMM diagnosis.

To distinguish between patients with SMM and MM, this study utilized recommendations from the IMWG and input from experts. Patients with the following characteristics indicating active MM at baseline were excluded: patients identified as having MM using the SLiM criteria (bone marrow infiltration ≥60%, FLC ratio ≥100 or >1 focal lesion >5 mm as determined by magnetic resonance imaging) and/or the CRAB criteria (hypercalcemia [serum calcium >0.25 mmol/l (>1 mg/dl) higher than the upper limit of normal or >2.75 mmol/l (>11 mg/dl)]; renal insufficiency (creatinine clearance <40 ml/min or serum creatinine >177 µmol/l [>2 mg/dl]); anemia (hemoglobin value of >20 g/l below the lower limit of normal, or a hemoglobin value < 100 g/l); bone lesions (≥1 osteolytic lesions on skeletal radiography or computed tomography [CT] or positron emission tomography-CT scan); or if the patient started MM treatment within 90 days of SMM diagnosis. Patients with light chain amyloidosis at baseline were excluded. In addition, patients who could not be classified into any risk group based on Mayo 20/2/20 criteria due to missing data were also excluded.

Patients who met the above identification criteria were classified into 2 groups (high risk and non–high risk [intermediate and low risk]) based on the Mayo 20/2/20 criteria [10]. High-risk SMM was defined as ≥2 of the following: FLC ratio >20 and <100, serum M protein >2 g/dl, or clonal BMPCs >20% to <60%. Due to the large degree of missing cytogenetic data (Table 1), it was not possible to evaluate data from patients with SMM risk stratified using the IMWG 2020 criteria that includes cytogenetic abnormalities.

**Table 1:** Baseline demographics and clinical characteristics^a,b^.

	Non–high risk (*n* = 324)	High risk (*n* = 174)	*P*-value
Age at SMM diagnosis, years			
Median (IQR)	65.0 (57–73)	67.0 (60–73)	0.256
Age group (*n*, %), years			
<18	0	0	
18–30	2 (0.6)	2 (1.1)	
31–40	13 (4.0)	0	
41–50	29 (9.0)	16 (9.2)	
51–60	68 (21.0)	34 (19.5)	
61–70	105 (32.4)	65 (37.4)	
71–80	83 (25.6)	52 (29.9)	
>80	24 (7.4)	5 (2.9)	
Female, *n* (%)	168 (51.9)	96 (55.2)	0.479
ECOG performance status, *n* (%)			0.102^c^
0	166 (51.2)	74 (42.5)	
1	139 (42.9)	91 (52.3)	
2	11 (3.4)	7 (4.0)	
3–4	4 (1.2)	0	
Missing	4 (1.2)	2 (1.1)	
BMPCs, %			<0.001^c^
Median (IQR)	15.0 (12.0–20.0)	29.3 (22.8–38.8)	
Missing, *n* (%)	1 (0.3)	4 (2.3)	
Involved: uninvolved serum FLC ratio			<0.001^c^
Median (IQR)	5.3 (2.1–12.1)	27.6 (11.8–44.2)	
Missing, *n* (%)	32 (9.9)	45 (25.9)	
Cytogenetics – t(4;14), *n* (%)			1.000^c^
Negative	94 (29.0)	72 (41.4)	
Positive	14 (4.3)	10 (5.7)	
Missing	216 (66.7)	92 (52.9)	
Cytogenetics – t(14;16), *n* (%)			0.453^c^
Negative	80 (24.7)	61 (35.1)	
Positive	1 (0.3)	3 (1.7)	
Missing	243 (75.0)	110 (63.2)	
Cytogenetics – gain(1q21), *n* (%)			1.000^c^
Negative	65 (20.1)	49 (28.2)	
Positive	44 (13.6)	34 (19.5)	
Missing	215 (66.4)	91 (52.3)	
Cytogenetics – del(17p13), *n* (%)			0.235^c^
Negative	98 (30.2)	65 (37.4)	
Positive	16 (4.9)	5 (2.9)	
Missing	210 (64.8)	104 (59.8)	
Immunoparesis of 2 immunoglobulins, *n* (%)			<0.001^c^
Yes	67 (20.7)	88 (50.6)	
No	214 (66.0)	62 (35.6)	
Missing	43 (13.3)	24 (13.8)	

*BMPC* bone marrow plasma cells, *ECOG* Eastern Cooperative Oncology Group, *FLC* free light chain, *IQR* interquartile range.

^a^Racial demographics were not recorded.

^b^Patients were risk-stratified using Mayo 20/2/20 criteria.

^c^Test performed on the non-missing cases only.

### Outcomes

#### Treatment regimens at the time of progression to MM

As there are many possible treatment regimens for MM, first line (1 L) treatment of patients after progression to active MM was categorized into mutually exclusive groups based on the agents contained in the regimen. Treatments containing anti-CD38 monoclonal antibodies were identified as anti-CD38-containing regimens; regimens containing proteasome inhibitors (PIs) but no anti-CD38 monoclonal antibodies were identified as PI-containing regimens; regimens containing immunomodulatory drugs (IMiDs) but no anti-CD38 monoclonal antibodies or PIs were identified as IMiD-containing regimens. For regimens not containing any of these 3 types of treatment, regimens with cytotoxic agents and corticosteroid-only regimens were differentiated. Furthermore, data from blinded clinical studies were also included in the RMG database, in which case regimens were categorized as such.

#### Key outcomes

Key outcomes included progression-free survival from SMM diagnosis to active MM diagnosis or death (PFS), progression-free survival from SMM diagnosis to progression on 1 L MM treatment or death (PFS2), and overall survival (OS). Specifically, among patients who received 1 L MM treatment, post-MM progression on 1 L MM treatment was also evaluated.

PFS was defined as the time from index date to MM diagnosis or death from any cause, whichever came first. If the patient was not deceased and did not have a diagnosis of MM based on the treating physician’s evaluation by the end of the follow-up period, patients were censored at the date that their last recording was made in the registry.

Among patients who received 1 L MM treatment, post-MM progression on 1 L MM treatment was defined as the time from the start of the 1 L MM treatment to death from any cause or progression, whichever came first. If the patient was not deceased or no such progression event was recorded after the start of 1 L treatment until the study cut-off date or lost to follow-up, whichever came first, patients were censored at the date that their last recording was made in the registry.

PFS2 was defined as the time from index date to documented progression on 1 L treatment for active MM or death from any cause, whichever came first. If a patient did not progress to MM and did not receive MM treatment or did not progress on 1 L MM treatment nor deceased, patients were censored at the date that their last recording was made in the registry.

OS was defined as the time from index date to death from any cause. Patients still alive were censored at the date that their last recording was made in the registry.

### Statistical analyses

Frequency and percentages were calculated for categorical variables, and continuous variables were summarized as means (standard deviation [SD]) and/or median (interquartile range [IQR]). Missing data were reported as a separate category.

The Kaplan-Meier method was used to estimate all time-to-event outcomes, including PFS, PFS2, and OS; log-rank tests were used to evaluate the difference in time-to-event endpoints of high-risk vs non–high-risk groups. Among patients who received 1 L MM treatment, post-MM progression on 1 L MM treatment was also assessed overall and stratified by the 1 L treatment category. Cox proportional hazards models were used to calculate the unadjusted and age-adjusted hazard ratio (HR) with 95% CI. All statistical tests were two-sided.

### Sensitivity analysis

As SMM diagnosis criteria have evolved over the past decade, sensitivity analyses were performed evaluating the outcomes among patients diagnosed with SMM more recently (from 2013 onward). It is noteworthy that the degree of missingness in the variables that were used to determine high risk was lower over this period of time and as such the percentage of patients that were excluded because of the missing values was lower.

## Results

Overall, 498 patients diagnosed with SMM were included in this study (Supplementary Table 1); 174 (34.9%) met the Mayo 20/2/20 criteria for high-risk SMM and 324 (65.1%) for non–high-risk SMM (including intermediate and low risk; Table 1). Median follow-up of the study was 63.9 months for high-risk patients and 66.7 months for non–high-risk patients. Four patients included in the analysis were diagnosed before 2000 (high risk, *n* = 1; non–high risk, *n* = 3; Supplementary Fig. 1).

### Baseline demographic and clinical characteristics

Median patient age at SMM diagnosis was similar between the risk groups, and most patients were between ages 51 and 80 years (Table 1). Sex distribution was not significantly different between the high-risk and non–high-risk groups. Eastern Cooperative Oncology Group (ECOG) performance status was also similar between the 2 risk groups, with most patients having an ECOG performance status of 0 or 1 (high risk, 94.8%; non–high risk, 94.1%). In the high-risk versus non–high-risk groups, median BMPC% were 29.3% versus 15.0% (*p* < 0.001) and FLC ratios were 27.6 versus 5.3 (*p* < 0.001), respectively. More than half of the patients with high-risk SMM (50.6%) had immunoparesis of 2 immunoglobulins compared with 20.7% of those with non–high-risk SMM (*p* < 0.001).

### First-line (1 L) MM treatment

In total, 57% of all patients with SMM received 1 L MM treatment, and this was higher in the high-risk group vs non–high-risk group (76.4% vs 46.6%, *p* < 0.001; Table 2). For both groups, these treatments were primarily PI- and/or IMiD-containing regimens (high-risk patients, 35.6% and 20.1%; non–high-risk patients, 25.0% and 13.8%, respectively). Fewer patients received an anti-CD38 containing regimen (high-risk patients, *n* = 5 [2.9%]; non–high-risk patients, *n* = 7 [2.2%]).

**Table 2:** First-line treatment after progression to MM.

	Non–high risk (*n* = 324)	High risk (*n* = 174)	*P*-value
First-line MM treatment, *n* (%)			<0.001
Yes	151 (46.6)	133 (76.4)	
No	173 (53.4)	41 (23.6)	
Patients by treatment type, *n* (%)			0.201
Anti-CD38	7 (2.2)	5 (2.9)	
Proteasome inhibitors	81 (25.0)	62 (35.6)	
Immunomodulatory agents	45 (13.9)	35 (20.1)	
Cytotoxic agents	17 (5.3)	27 (15.5)	
Corticosteroids	1 (0.3)	3 (1.7)	
Blinded clinical study	0	1 (0.6)	

*MM* multiple myeloma, *SMM* smoldering multiple myeloma.

### Time-to-event outcomes

**Fig. 1:**
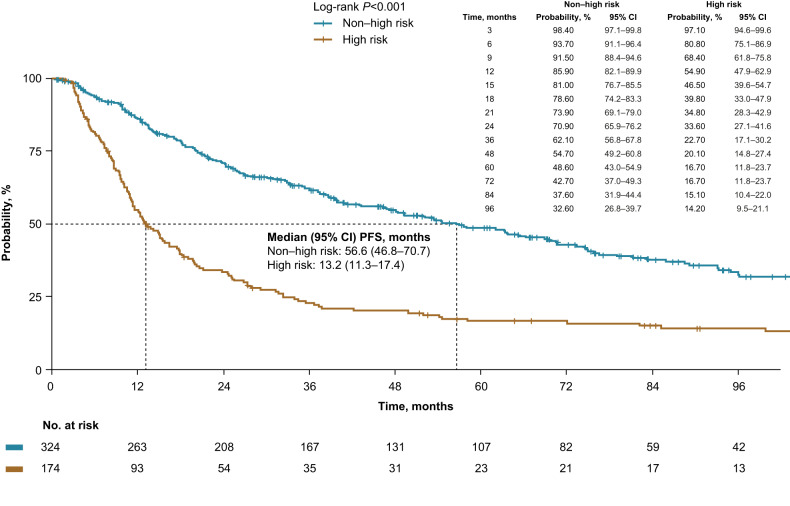
Progression-free survival from SMM diagnosis to active MM diagnosis (PFS). Patients were risk stratified using Mayo 20/2/20 criteria. CI confidence interval, MM multiple myeloma, SMM smoldering multiple myeloma.

Median PFS from SMM diagnosis to active MM diagnosis was 33.3 months overall and was significantly shorter in high-risk patients than non–high-risk patients (high risk vs non–high risk, 13.2 vs 56.6 months, *p* < 0.001; Fig. 1). High-risk patients had a higher risk of progression or death compared with non–high-risk patients (Table 3).

**Table 3:** Adjusted and unadjusted hazard ratios for outcomes in high-risk vs non–high-risk patients.

	Age-adjusted		Unadjusted	
Outcome	HR (95% CI)	*P*-value	HR (95% CI)	*P*-value
PFS	2.47 (1.98–3.09)	<0.001	2.52 (2.01–3.15)	<0.001
Post-MM progression on 1 L MM treatment	0.90 (0.66–1.22)	0.494	0.87 (0.65–1.18)	0.368
PFS2	1.74 (1.35–2.26)	<0.001	1.73 (1.34–2.23)	<0.001
OS	1.52 (1.13–2.04)	0.006	1.45 (1.08–1.95)	0.013

*1L* first line, *MM* multiple myeloma, *OS* overall survival, *PFS* progression-free survival from SMM diagnosis to active MM diagnosis or death, *PFS2* progression-free survival from SMM diagnosis to progression on 1 L MM treatment or death.

**Fig. 2:**
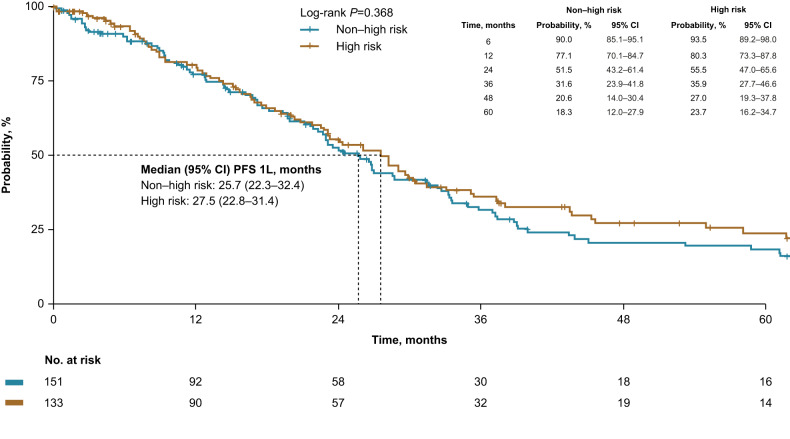
Post-MM progression on 1 L MM treatment. Patients were risk stratified using Mayo 20/2/20 criteria. CI confidence interval, MM multiple myeloma.

In patients who received 1 L MM treatment (*n* = 284), median post-MM progression on 1 L treatment was 26.6 months and was similar between risk groups (high risk vs non–high risk, 27.5 vs 25.7 months, *p* = 0.368; Fig. 2) and the risk of progression or death was similar between groups (Table 3). In the analysis stratified by 1 L treatment, only the PI-containing regimens, IMiD-containing regimens, and cytotoxic regimen strata had enough patients to be meaningfully evaluated, and no significant difference in post-MM progression on 1 L treatment was found between risk groups for the 3 strata.

**Fig. 3:**
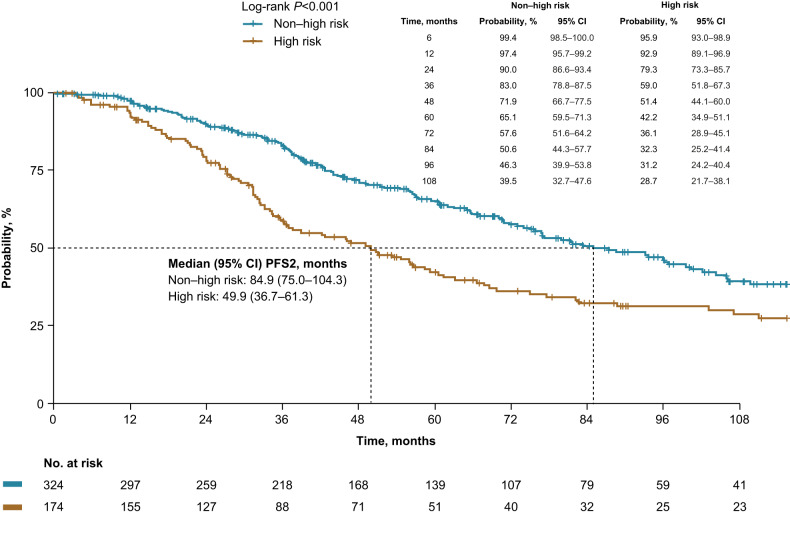
Progression-free survival from SMM diagnosis to progression on 1 L MM treatment (PFS2). Patients were risk stratified using Mayo 20/2/20 criteria. CI confidence interval, MM multiple myeloma, SMM smoldering multiple myeloma.

Median PFS2 was 71.8 months and was significantly shorter in high-risk patients (high risk, 49.9 months vs non–high risk, 84.9 months; *p* < 0.001; Fig. 3) and compared with non–high-risk patients, high-risk patients had an increased risk of progression or death (Table 3).

In total, 35.9% of patients died during the follow-up period; 80 (46.0%) high-risk and 99 (30.6%) non–high-risk patients. Causes of death (myeloma- or non-myeloma-related) are shown in Table 4. Approximately half of the deaths in the high-risk group and about a third in the non-high-risk group resulted from a myeloma-related event.

**Table 4:** Causes of death in the total population (n corresponds to the number of deaths).

Cause of death	Non–high risk (*n* = 99)	High risk (*n* = 80)
Myeloma-related	34 (34.3%)	38 (47.5%)
Non-myeloma-related	60 (60.6%)	37 (46.2%)
Unknown	5 (5.1%)	5 (6.2%)

**Fig. 4:**
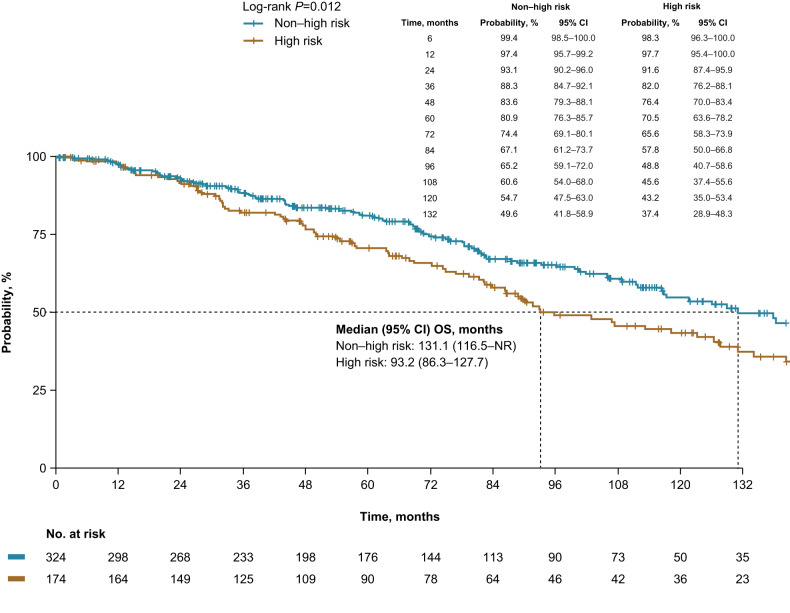
Overall survival from SMM diagnosis. Patients were risk stratified using Mayo 20/2/20 criteria. CI confidence interval, SMM smoldering multiple myeloma.

Median OS was 121.7 months for the entire patient population (high risk vs non–high risk, 93.2 vs 131.1 months, *p* = 0.012; Fig. 4). Compared with non–high-risk patients, high-risk patients had a higher risk of death (Table 3). Yearly survival rates were similar until approximately 24 months of follow-up. Differences in OS rates between the high-risk and non–high-risk patients were observed after about 36 months and were >10% apart at 60 and 120 months (70.5% vs 80.9% and 43.2% vs 54.7%, respectively).

#### Sensitivity analyses in patients diagnosed with SMM from 2013 onward

Overall, 249 of the enrolled patients were diagnosed with SMM from 2013 onward (high risk, *n* = 72; non–high risk, *n* = 177). Median follow-up of these patients was shorter than the main analysis (high-risk patients, 38.5 months; non–high-risk patients, 38.9 months). Baseline demographics and clinical characteristics of these patients are shown in Supplementary Table 2.

In total, 47.0% of patients diagnosed with SMM from 2013 onward received first-line MM treatment during the study period; this was higher in the high-risk than the non–high-risk group (70.8% vs 37.3%, respectively; Supplementary Table 3). For both risk groups, these treatments were primarily PI- and/or IMiD-containing regimens. Of the 12 patients receiving anti-CD38 treatment in the full population, all but 1 patient in the non–high-risk group were diagnosed with SMM from 2013 onward.

Like the results from the main analysis, median PFS from SMM diagnosis to active MM diagnosis in patients diagnosed from 2013 onward was shorter in high-risk patients (Supplementary Fig. 2), with a greater risk of progression vs non–high-risk patients (Supplementary Table 4). For patients who received 1 L MM treatment, median post-MM progression on 1 L treatment (Supplementary Fig. 3) and the risk of progression or death was similar between risk groups (Supplementary Table 4). However, unlike the main analyses, median PFS2 in patients diagnosed from 2013 onward (Supplementary Fig. 4) was not significantly different between high-risk and non–high-risk patients, although median PFS2 was numerically shorter in high-risk patients. The risk of progression or death was similar between risk groups (Supplementary Table 4). In total, 24.1% of patients diagnosed from 2013 onward died during the follow-up period; 20 (27.8%) high-risk and 40 (22.6%) non–high-risk patients. Causes of death (myeloma- or non-myeloma-related) are shown in Supplementary Table 5. A little less than half of the deaths in the high-risk group and a quarter in the non-high-risk group resulted from a myeloma-related event. Median OS was not reached in patients diagnosed from 2013 onward (Supplementary Fig. 5).

## Discussion

These real-world data from the RMG database demonstrate that patients with high-risk SMM (defined using the Mayo 20/2/20 criteria) were more likely to progress to active MM than those with non–high-risk SMM. In addition, in the full population, patients with high-risk SMM progressed more quickly to active MM compared to patients with non-high-risk SMM. The median PFS observed in patients with high-risk SMM in this study (13.2 months) was shorter than reported in previous studies [18, 19]. Patients with high-risk SMM also had worse OS than those with non–high-risk SMM. Yearly survival rates were similar between the risk groups until approximately 24 months, with differences in OS observed after 36 months. High-risk patients also had a shorter PFS2 than non–high-risk patients. However, PFS2 could have been impacted by the type of 1 L MM treatment.

NCCN have recognized these unmet needs for patients with SMM and their guidelines currently recommend actively monitoring patients with SMM at 3–6 months intervals or enrolling patients with SMM in clinical trials regardless of risk [14]. For selected high-risk patients, the NCCN guidelines also recommend the use of off-label treatment with lenalidomide. Findings from this study support the clinical unmet needs of patients with high-risk SMM and their continued need for better survival outcomes.

Phase 3 clinical trials, such as QuiReDex and E3A06, have established the benefit of early intervention in improving outcomes in patients with high-risk SMM [18, 20]. In the QuiReDex study, treatment of high-risk patients with SMM with lenalidomide plus dexamethasone (Rd) delayed progression to active MM and improved OS compared with observation [20]. For the main analysis, high risk was defined as either bone marrow plasma cell infiltration of at least 10% or presence of monoclonal component (IgG ≥3 g/dl or IgA ≥2 g/dl, or Bence Jones proteinuria >1 g/24 h), or both, plus at least 95% phenotypically aberrant plasma cells in the bone marrow plasma cell compartment with immunoparesis (reductions in one or 2 uninvolved immunoglobulins of >25% compared with normal values). A post hoc analysis of QuiReDex, which evaluated outcomes in high-risk patients, defined specifically using the Mayo 2007 criteria (serum M protein ≥30 g/L and BMPC ≥ 10%) showed that time to progression was significantly improved in the treatment (lenalidomide and dexamethasone) versus the observation group (HR, 0.21 [95% CI: 0.10–0.40]; *p* < 0.0001) [20]. A long-term follow-up of the QuiReDex trial at a follow-up of 12.5 years confirmed that early treatment with Rd for high-risk SMM resulted in sustained improvements in time to progression and OS [21]. Results of the E3A06 trial in patients with high-risk SMM defined using the Mayo 20/2/20 criteria demonstrated improvement in PFS and delay in development of end-organ damage with early lenalidomide monotherapy versus observation [18]. Other ongoing phase 3 clinical trials, such as AQUILA, DETER-SMM, and ITHACA, are currently evaluating further therapeutic regimens for the treatment of high-risk SMM, including subcutaneous daratumumab monotherapy, daratumumab plus lenalidomide and dexamethasone, and isatuximab plus lenalidomide and dexamethasone, respectively [22–24].

The results from this real-world study demonstrated that high-risk patients with SMM were more than 2.5 times more likely to progress to active MM. While the results from the sensitivity analysis in patients diagnosed with SMM from 2013 onward did not show statistically significant differences in PFS2 between the 2 groups, which may potentially be due to the data being more immature, PFS2 was numerically shorter in the high-risk versus non–high-risk group. Median OS was not reached in either risk group in patients diagnosed after 2013.

Compared with other real-world studies of patients with SMM who were defined as high-risk using the Mayo 20/2/20 criteria, median follow-up of this study was longer (approximately 65 months from SMM diagnosis) than that in a large multi-center study by Mateos et al. [11] (median follow-up, 36 months) and marginally shorter than that of a study by Lakshman et al. [10] from the Mayo Clinic Dysproteinemia database (median follow-up, 74.8 months).

Data used in this study were compiled from patients from multiple centers in the Czech Republic, that provided the estimates of long-term clinical characteristics and outcomes. Another strength of this disease registry was access to almost complete death records, which allowed for consistency, reliability, and accuracy of the available data when evaluating mortality. In addition, to our knowledge, this is the first study to evaluate PFS2 in this patient population. This current study is of great value considering the low number of publications on SMM and the lack of disease quality registries that gather clinical information among patients with SMM.

As with other retrospective observational studies in patients with SMM, one of the limitations of this study is the difficulty in diagnosing patients due to the asymptomatic nature of the condition. As such, these results may not reflect the entire population of people with SMM in the Czech Republic. In addition, because patients were selected from one country only, the generalizability of the results outside of the Czech population is limited and future studies in a larger population of patients are warranted to confirm the findings of this study. MRIs were not performed on all patients and as such it is possible that some patients classified as having high risk SMM may have progressed to active MM at the start of the study potentially contributing to the short PFS observed in the high-risk patients in this study. As the registry was established in 2007, data before then had been entered retrospectively and may not fully represent all patients prior to that date. Other risk stratification models such as the IMWG 2020 model can help assess the consistency of risk assessment on high-risk patients; however, it was not possible to evaluate cytogenetic profiles due to a lack of such data in most patients with SMM, and as such high-risk definitions requiring this data could not be assessed. An analysis of patients who were treated for SMM versus those who were not treated was not possible as these data were not specifically recorded in the RMG database and only those patients who progressed and were treated for MM were noted as such. Data from patients receiving treatment in clinical trials were included, and it should be noted that some of these treatments such as anti-CD38 antibodies are not currently approved nor reimbursed for 1 L treatment of MM in the Czech Republic. In this study, it was not possible to evaluate differences in survival between treated and non-treated patients who progressed to MM as the overwhelming majority of those who did progress received MM treatment, the initiation of which generally followed a standard time course (data not shown). Finally, OS may be influenced by treatments reimbursed in the Czech Republic at the time of the study.

## Conclusion

These analyses add to the growing body of evidence that risk stratification can be used to identify patients with SMM most likely to have worse outcomes. Additional studies are needed to assess the implications of SMM management on MM outcomes and to evaluate outcomes in patients treated early for SMM versus at the time of MM diagnosis. Information on early intervention in patients with high-risk SMM to delay or perhaps even prevent the onset of active MM will be critical for patients, clinicians, and decision-makers.

### Supplementary information

Supplementary Materials

## Data Availability

The datasets generated during and/or analyzed during the current study are available from the corresponding author on reasonable request.
