# Supplementary material for: Clinical characteristics and outcomes in risk-stratified patients with smoldering multiple myeloma: data from the Czech Republic Registry of Monoclonal Gammopathies
Source: Blood Cancer J. 2023 Sep 27;13(1):153. doi: 10.1038/s41408-023-00906-7 (PMC10522616; doi:10.1038/s41408-023-00906-7)
Supplement: Supplementary file 1 — Supplementary Materials [file 41408_2023_906_MOESM1_ESM.pdf]

Supplemental Materials

Supplemental Table 1. Patient attrition.

Step	Inclusion/Exclusion Criteria	N	%
1	Patients meeting one of the following SMM criteria at the time of diagnosis (from 1980 to November 2021)<br>• clonal BMPCs ≥10%<br>• serum M protein (IgG or IgA) ≥30g/l<br>• urinary M protein ≥500 mg per 24 h<br>Index date: date of SMM diagnosis form	6 476	100.0%
2	Of step 1, exclude patients with one of the following MM defining events on the SMM diagnosis form (or prior forms)<br>• Hypercalcemia: serum calcium >0.25 mmol/l (>1 mg/dl) higher than the upper limit of normal or >2.75 mmol/l (>11 mg/dl)<br>• Renal insufficiency: creatinine clearance <40 ml per min or serum creatinine >177 µmol/l (>2 mg/dl)<br>• Anemia: hemoglobin value of >20 g/l below the lower limit of normal, or a hemoglobin value <100 g/l<br>• Bone lesions: one or more osteolytic lesions on skeletal radiography, CT, or PET-CT<br>• clonal BMPCs ≥60%<br>• involved: uninvolved serum FLC ratio ≥100	897	13.9%
3	Of step 2, exclude patients who die or start treatment within 90 days of index date	609	9.4%
4	Of step 3, exclude patients with missing date for survival analysis	583	9.0%
5	Of step 4, patients fulfilling either C) or D):<br>C) At least 2 of FLC, BMPC and M protein is missing<br>D) EXACTLY 1 is TRUE from the following:<br>• BMPC is missing<br>• FLC is missing<br>• M protein is missing<br>AND EXACTLY 1 is TRUE from the following:<br>• BMPC ≤20%<br>• M protein ≤20 g/l<br>• FLC ≤20<br>AND EXACTLY 1 is TRUE from the following:<br>• BMPC >20%<br>• M protein >20 g/l<br>• FLC >20	498	7.7%
6	**MAYO high-risk**<br>Of step 5, patients who fulfill at least 2 of the following criteria:<br>• Serum M protein >20 g/l<br>• Serum involved:uninvolved FLC ratio >20 and <100<br>• Clonal BMPCs >20% to <60%	174	2.7%

BMPC bone marrow plasma cell; *CT* computed tomography; *FLC* free light chain; *IgA* immunoglobulin A; *IgG* immunoglobulin G; *MM* multiple myeloma; *PET-CT* positron emission tomography-computed tomography; *SMM* smoldering multiple myeloma.

Supplemental Table 2. Baseline demographics and clinical characteristics of patients diagnosed from 2013 onward.[a,b]

	**Non–high risk (*n* = 177)**	**High risk (*n* = 72)**	***P*-value**
Age at SMM diagnosis, years			
Median (IQR)	68.0 (60–73)	69.0 (62–74)	0.167
Age group, years, n (%)			
<18	0	0	
18–30	1 (0.6)	0	
31–40	7 (4.0)	0	
41–50	15 (8.5)	5 (6.9)	
51–60	24 (13.6)	8 (11.1)	
61–70	65 (36.7)	30 (41.7)	
71–80	50 (28.2)	28 (38.9)	
>80	15 (8.5)	1 (1.4)	
Female, n (%)	86 (48.6)	37 (51.4)	0.689
ECOG performance status, n (%)			0.413[c]
0	106 (59.9)	42 (58.3)	
1	60 (33.9)	29 (40.3)	
2	7 (4.0)	1 (1.4)	
3–4	3 (1.7)	0	
Missing	1 (0.6)	0	
BMPCs, %			<0.001[c]
Median (IQR)	15.0 (12.0–20.0)	26.0 (22.5–40.0)	
Missing, n (%)	1 (0.6)	3 (4.2)	
Involved: uninvolved serum FLC ratio			<0.001[c]
Median (IQR)	5.4 (2.3–13.0)	29.0 (16.7–41.9)	
Missing, n (%)	6 (3.4)	3 (4.2)	
Immunoparesis of 2 immunoglobulins, n (%)			<0.001[c]
Yes	41 (23.2)	35 (48.6)	
No	113 (63.8)	28 (38.9)	
Missing	23 (13.0)	9 (12.5)	

[a]Racial demographics were not recorded. [b]Patients were risk stratified using Mayo 20/2/20 criteria. [c]Test performed on the non-missing cases only.

BMPC bone marrow plasma cells; *ECOG* Eastern Cooperative Oncology Group; *FLC* free light chain; *IQR* interquartile range.

Supplemental Table 3. First-line treatment after progression to MM in patients diagnosed with SMM from 2013 onward.

	**Non–high risk (*n* = 177)**	**High risk (*n* = 72)**	***P*-value**
First-line MM treatment, n (%)			<0.001
yes	66 (37.3)	51 (70.8)	
no	111 (62.7)	21 (29.2)	
Patients by treatment type, n (%)			0.408
Anti-CD38	6 (3.4)	5 (6.9)	
Proteasome inhibitors	46 (26.0)	40 (55.6)	
Immunomodulatory agents	12 (6.8)	4 (5.6)	
Cytotoxic agents	2 (1.1)	1 (1.4)	
Corticosteroids	0	1 (1.4)	

MM multiple myeloma; *SMM* smoldering multiple myeloma.

Supplemental Table 4. Adjusted and unadjusted hazard ratios for outcomes in non–high-risk vs high-risk patients diagnosed with SMM from 2013 onward.

	**Age-adjusted**		**Unadjusted**	
**Outcome**	**HR (95% CI)**	***P*-value**	**HR (95% CI)**	***P*-value**
PFS	2.45 (1.73 –3.46)	<0.001	2.55 (1.81 –3.61)	<0.001
Post-MM progression on 1L MM treatment	0.74 (0.44 –1.26)	0.272	0.76 (0.46 –1.27)	0.302
PFS2	1.43 (0.92 –2.24)	0.115	1.44 (0.92 –2.24)	0.110
OS	1.47 (0.84 – 2.55)	0.174	1.25 (0.73 – 2.13)	0.422

1L; first line; *MM* multiple myeloma; *OS* overall survival; *PFS* progression-free survival from SMM diagnosis to active MM diagnosis or death; *PFS2* progression-free survival from SMM diagnosis to progression on 1L MM treatment or death.

Supplemental Table 5. Causes of death in the population from 2013 onward (n corresponds to the number of deaths).

**Cause of death**	**Non–high risk (*n* = 40)**	**High risk (*n* = 20)**
Myeloma-related	10 (25.0%)	9 (45.0%)
Non-myeloma-related	26 (65.0%)	9 (45.0%)
Unknown	4 (10.0%)	2 (10.0%)

Supplemental Figure 1. Patients enrolled by calendar year. Patients were risk stratified using Mayo 20/2/20 criteria.

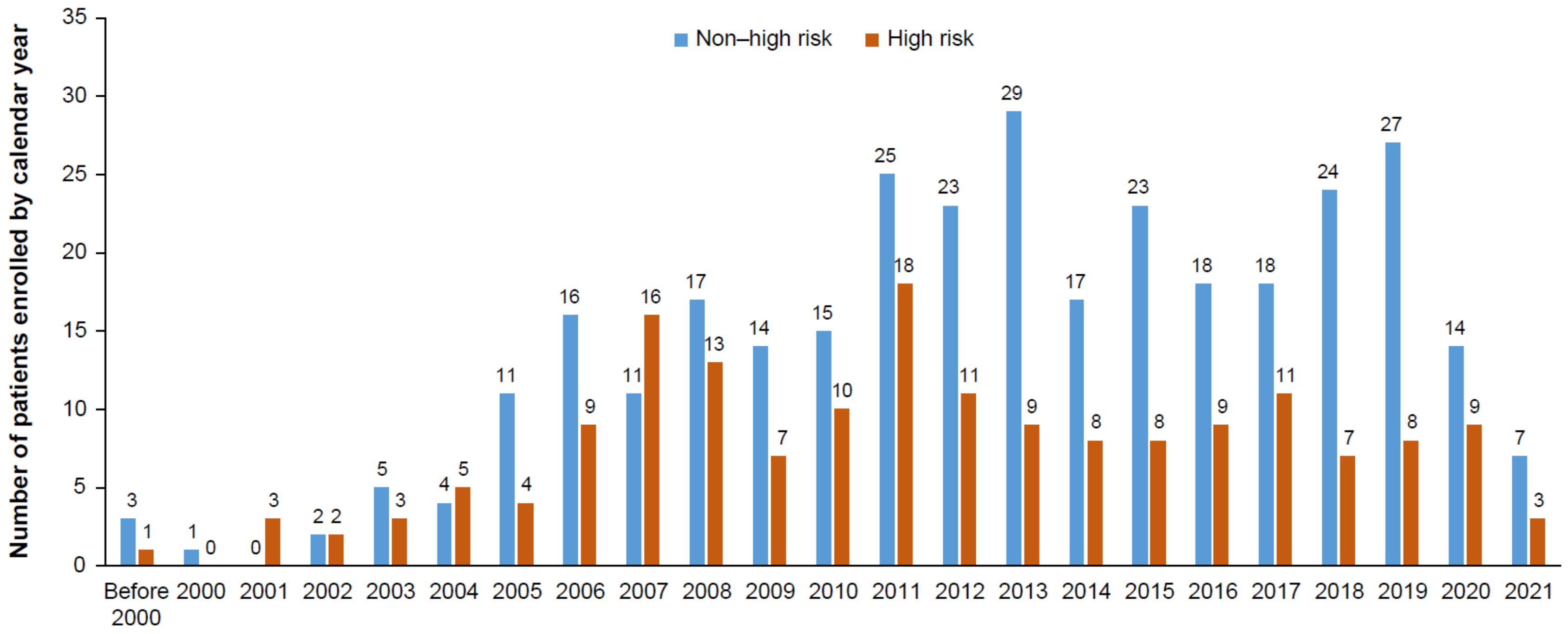

Supplemental Figure 2. Progression-free survival in patients diagnosed with SMM from 2013 onward.[a] Patients were risk stratified using Mayo 20/2/20 criteria.

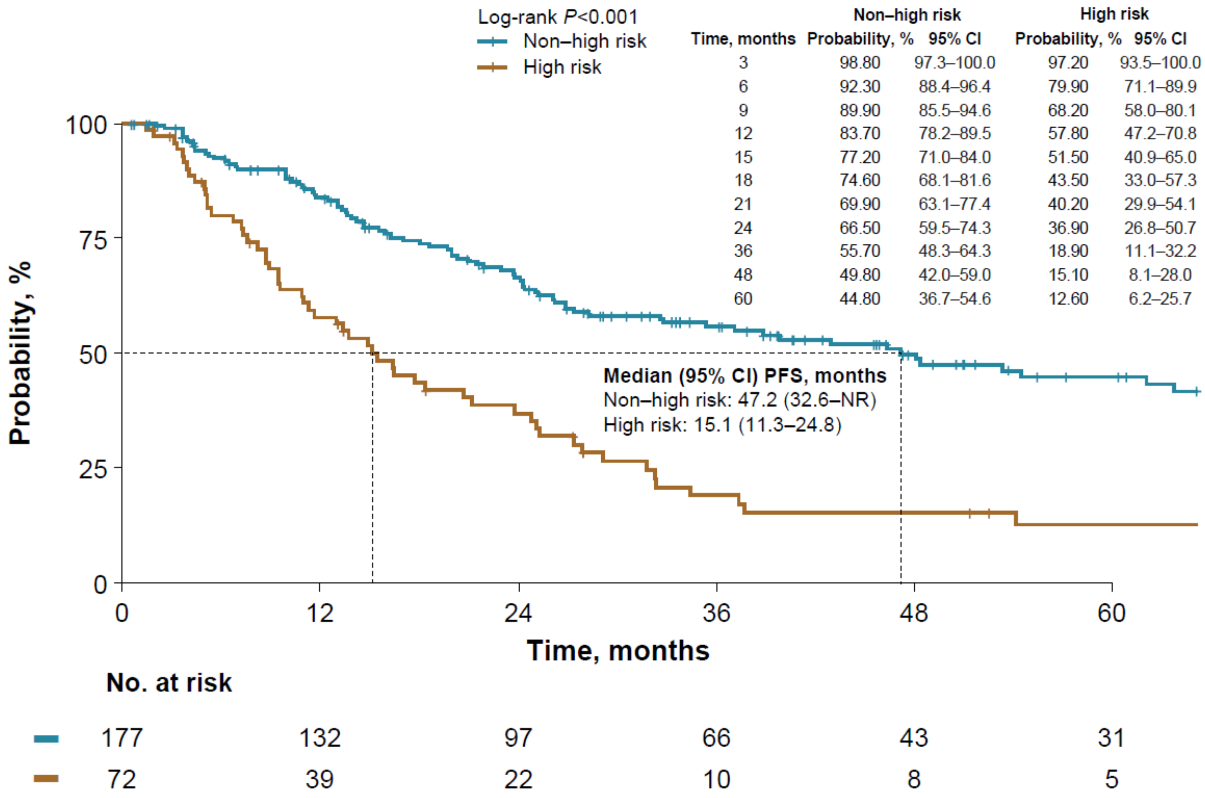

[a]From SMM diagnosis.

CI confidence interval; *MM* multiple myeloma; *SMM* smoldering multiple myeloma.

Supplemental figure 3. Post-MM progression on 1L MM treatment in patients diagnosed with SMM from 2013 onward. Patients were risk stratified using Mayo 20/2/20 criteria.

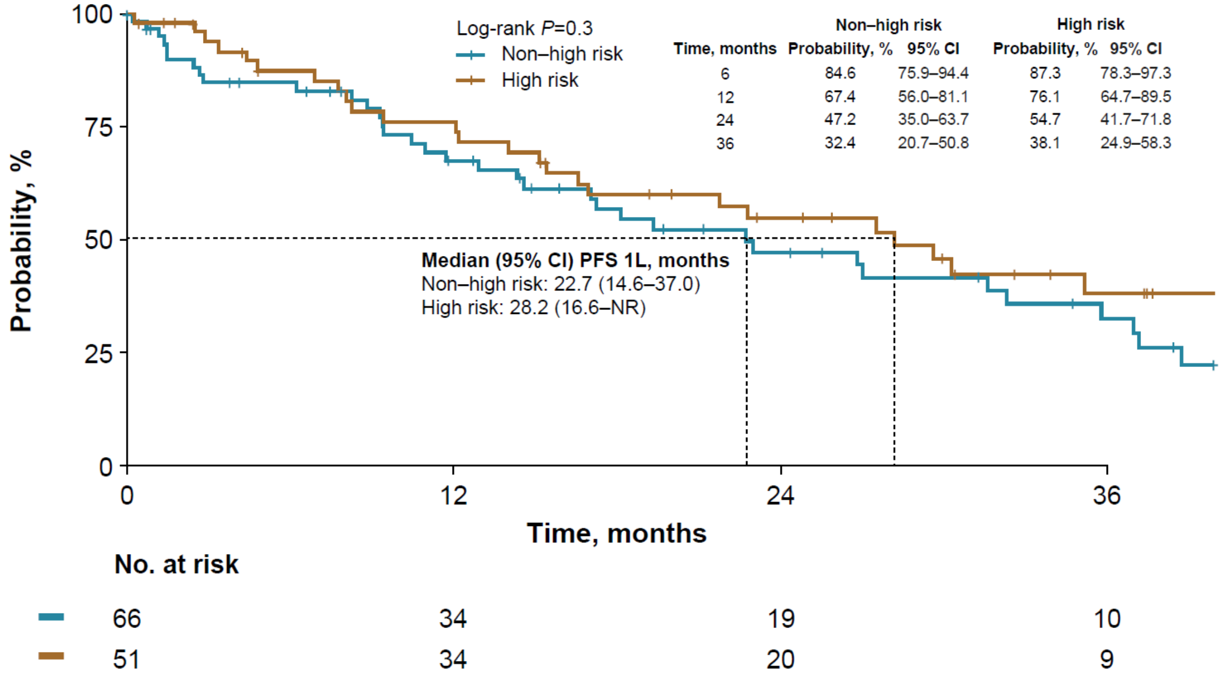

CI confidence interval; *SMM* smoldering multiple myeloma.

Supplemental Figure 4. Progression-free survival from SMM diagnosis to progression on 1L MM treatment (PFS2) in patients diagnosed with SMM from 2013 onward.[a] Patients were risk stratified using Mayo 20/2/20 criteria.

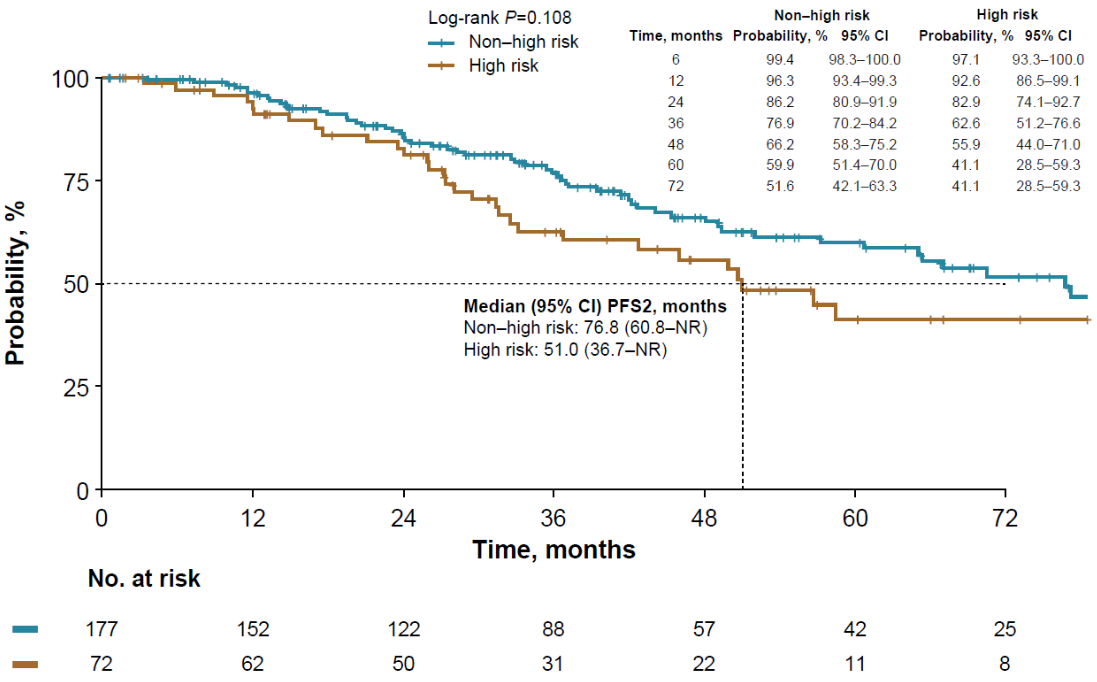

[a]From SMM diagnosis date.

CI confidence interval; *SMM* smoldering multiple myeloma.

Supplemental Figure 5. Overall survival from SMM diagnosis data in patients diagnosed with SMM from 2013 onward. Patients were risk stratified using Mayo 20/2/20 criteria.

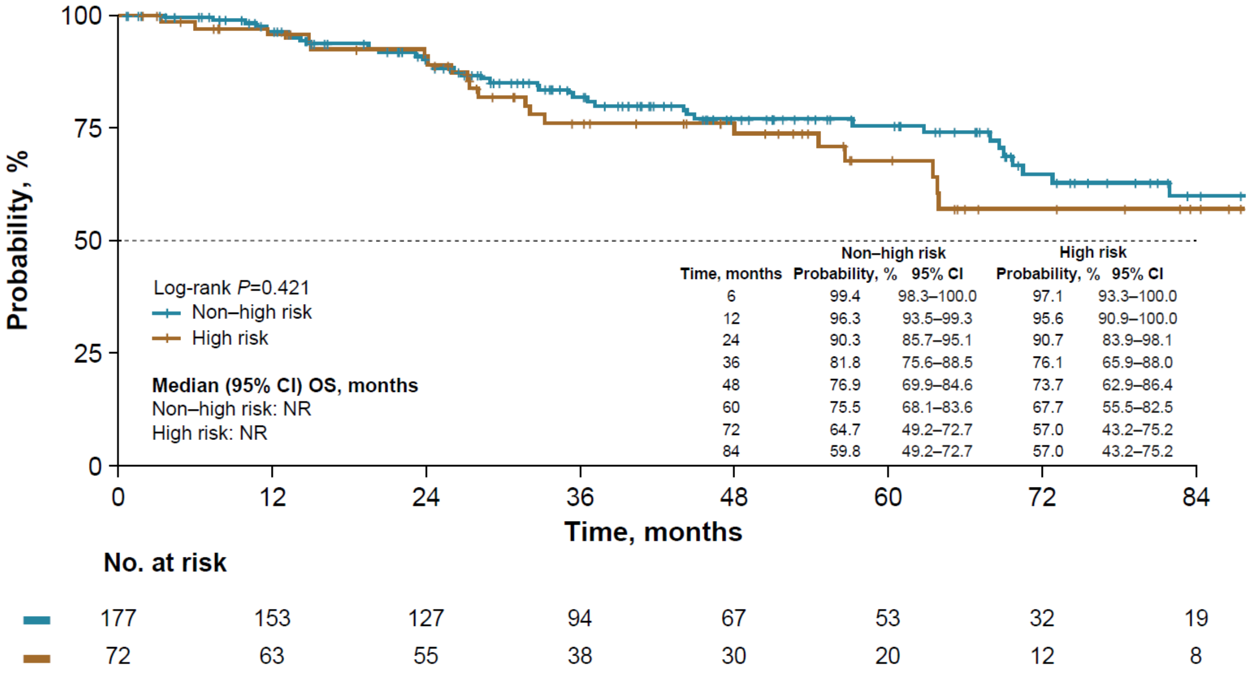

CI confidence interval; *SMM* smoldering multiple myeloma.
